# Immune Checkpoint Inhibitors in Tumors Harboring Homologous Recombination Deficiency: Challenges in Attaining Efficacy

**DOI:** 10.3389/fimmu.2022.826577

**Published:** 2022-02-08

**Authors:** Saulo Brito Silva, Carlos Wagner S. Wanderley, Leandro Machado Colli

**Affiliations:** ^1^ Department of Medical Imaging, Hematology, and Oncology, Ribeirao Preto Medical School, University of Sao Paulo, Ribeirao Preto, Brazil; ^2^ Department of Pharmacology, Ribeirao Preto Medical School, University of Sao Paulo, Ribeirao Preto, Brazil

**Keywords:** immune checkpoint inhibitors, homologous recombination, DNA damage repair, mismatch repair, oncology

## Abstract

Cancer cells harbor genomic instability due to accumulated DNA damage, one of the cancer hallmarks. At least five major DNA Damage Repair (DDR) pathways are recognized to repair DNA damages during different stages of the cell cycle, comprehending base excision repair (BER), nucleotide excision repair (NER), mismatch repair (MMR), homologous recombination (HR), and non-homologous end joining (NHEJ). The unprecedented benefits achieved with immunological checkpoint inhibitors (ICIs) in tumors with mismatch repair deficiency (dMMR) have prompted efforts to extend this efficacy to tumors with HR deficiency (HRD), which are greatly sensitive to chemotherapy or PARP inhibitors, and also considered highly immunogenic. However, an in-depth understanding of HRD’s molecular underpinnings has pointed to essential singularities that might impact ICIs sensitivity. Here we address the main molecular aspects of HRD that underlie a differential profile of efficacy and resistance to the treatment with ICIs compared to other DDR deficiencies.

## Introduction

The central DNA Damage Repair (DDR) pathways comprise base excision repair (BER), nucleotide excision repair (NER), mismatch repair (MMR), homologous recombination (HR), and non-homologous end joining (NHEJ), which are collectively responsible for repairing DNA damages during different stages of the cell cycle (1). In tumor cells, defects on DRR pathways, by one hand, works as a source of genetic diversity and mutations that are beneficial for tumor evolution. On the other hand, it exposes the tumor cell to fragilities not observed in normal cells. In this context, the functional status of the DDR system has long been recognized as a biomarker for a broad range of treatments (2).

Different therapies could take advantage of DDR pathways’ defects to induce additional tumor genetic structural damage, as with radiotherapy, cytotoxic chemotherapies, or targeted DNA repair mechanisms such as PARP inhibitors, to enhance tumors cells’ lethality (3). In addition, recently, mismatch repair-deficient (dMMR) tumors have consistently been shown to harbor greater immunogenicity and be highly effective to immune checkpoint inhibitors (ICIs) (4, 5). Consequently, dMMR granted accelerated approval by the FDA to ICIs agnostic use to treat advanced solid tumors (6).

From this point onwards, understanding whether this effect also extends to other DDR pathways started to be deeply investigated. Although any type of DDR dysfunction can lead to the accumulation of tumoral mutations, there is a wide variety in burden and type of mutations, depending on the DNA level each repair mechanism actuates (7). However, the impact of those different pattern of mutations on immunogenicity and, consequently, on the response to immunotherapy is still a matter of debate.

The benefits achieved with ICIs in tumors with dMMR have prompted efforts to extend this efficacy to tumors with HRD, which are highly sensitive to chemotherapy or PARP inhibitors and expected to be highly immunogenic. Nonetheless, molecular underpinnings of HR defects have pointed to singularities that might impact antitumor immune response and ICIs effectiveness. This review will summarize the main molecular aspects of HRD that underlie a differential profile of efficacy and resistance to the treatment with ICIs compared to other DDR deficiencies.

### DDR in Current Clinical Practice

#### Mismatch Repair

The most significant evidence linking DNA repair deficiency with ICIs activity stems from tumors with a deficiency in mismatch repair (MMR) (dMMR). Roughly 18% of endometrial cancers, 11% of ovarian cancers, and 4% of metastatic colorectal cancer present with mutations or epigenetic silencing in genes comprising the MMR system (8). In a phase II clinical trial evaluating pembrolizumab in a set of treatment-refractory dMMR tumors, the response rates were as high as 40% to 70% (9). The studies Checkmate 142 (10) and Keynote 164 (11), which evaluated nivolumab and pembrolizumab, respectively, led to ICIs’ first approval, in dMMR tumors, for colorectal cancer previously submitted to chemotherapy. In addition, the Keynote 177 study (12) currently supports pembrolizumab use in the first-line setting of colorectal cancers. Finally, the Keynote 158 study (6) led to pembrolizumab approval for previously treated dMMR tumors irrespective of histology. Such an efficacy led to MMR status evaluation in current clinical practice for a broad set of other tumor histologies wherein this DDR deficiency can also be noticed, such as stomach, biliary tract, pancreas, prostate, and small intestine cancer (13).

#### Homologous Recombination

Homologous recombination (HR) is the most likely DDR mechanism found when considering a non-selected histology-based population (14). It is a crucial pathway to repair double-strand DNA breaks due to its error-free repairing system that relies on an intact sister chromatid instead of the non-homologous end joining (NHEJ) process (7). The incidence of pathogenic HRD varies according to histology, staging, and previous treatment burden (15). Notwithstanding, HRD is currently most recognized in tumors for which PARP inhibitors are currently approved based on a biomarker-guided *BRCA* or HR loss of function: ovarian cancer (40-50% with HRD), prostate cancer (20-25%), breast cancer (18%), and pancreatic cancer (12%) (16–20). Recently, many other malignancies were also shown to have a high incidence of HRD, such as endometrial (34%), biliary tract (28%), bladder (23%), hepatocellular (20%), and gastroesophageal cancer (20%) (14).

In contrast to the high clinical efficacy of ICIs in MMR deficient tumors, the clinical benefits are not consistent with an HRD. In phase II KEYNOTE 100 study, response rates with pembrolizumab in patients with advanced ovarian cancer were less than 10% among those harboring an HRD, with no statistical difference found when comparing BRCA-mutated versus wild-type counterparts (21). Despite other HRD genes being currently tested in ovarian cancer through NGS platforms, no prospective clinical data have evaluated their differential effectiveness, such that all available clinical data stem from BRCA-mutated tumors. Moreover, in phase III Keynote 119, patients with previously treated triple-negative breast cancers - approximately 50% of whom have HRD – derived no benefit from pembrolizumab compared to chemotherapy concerning response rate or survival (22). Although this study was not designed to evaluate patients with breast cancer having HRD specifically, both those ovarian and breast early clinical data shed light on a significant difference in clinical efficacy compared to what is seen early on with ICIs for dMMR tumors. In addition, those evidence has ultimately contributed to shifting strives for various ICIs combinations that are now undergoing prospectively to overcome such immune restoration mitigation – through anti-PD-1/PD-L1 –, which is taking place in the presence of HRD and remain underrecognized.

### DDR and Immunogenicity

Deficient DDR processes that predispose to genetic alterations at the DNA sequence level, such as in dMMR, have the highest potential to elicit antigenicity due to the vast number of mutation-associated neoantigens (23). Since it has been shown that only a tiny fraction of predicted neo-epitopes are presented through MHC-I to enable T-cell responses (24, 25), it seems likely that tumors with a higher number of tumor mutation burden (TMB) are more likely to present with neoantigens that effectively stimulate the immune system (26).

Extensive mutational assessments have demonstrated enrichment in single- and multi-nucleotide variants (SNVs and MNVs) in tumors with dMMR, resulting in a high TMB, generally higher than 17 mutations/Mb (27). In the rare inherent genetic condition of bi-allelic germline dMMR, tumors can display >250 mutations/Mb (28). Due to dMMR tumors’ high immunogenicity, ICIs are substantially effective in various settings, thus warranting approval on an agnostic indication basis. Regarding other hypermutated tumors, yet non-dMMR, a TCGA analysis has shown that somatic mutations in polymerase epsilon (POLE) or delta POLD1 also comprise a DDR deficient group with high TMB (29). Like in dMMR, impairment in the proofreading capability of *POLE* and *POLD1* leads to genetic alterations at the DNA sequence level. Pathogenic somatic mutations in the proofreading exonuclease domain of *POLE* confer similar phenotypes regardless of the tumor tissue type, resulting in a large mutation rate, especially TCT>TAT and TCG>TTG transversions and, more rarely, concomitant microsatellite instability (30). Although somatic mutations in POLE have been identified in 2–8% of colorectal cancer and 7–15% of endometrial carcinoma (31), there are little data available reporting ICIs efficacy in these DDR populations due to their low incidence and the absence of systematic screening in daily practice (32, 33). Interestingly, extensive mutational profiling of 21.074 patients from 23 cancer types and subtypes suggested that *POLE/POLD1* mutation was not independently associated with survival to ICIs treatment after adjusting for TMB. The study concludes that mutations in the proofreading domain of *POLE/POLD1* are more likely to result in DNA repair defects featuring extremely high TMB, which contribute to more significant benefits from ICI treatment (34).

Tumors with HRD also have a higher mutational load and predicted neo-epitopes than those without DDR deficiencies (35). Intriguingly, when considering patients with high TMB tumors that are not MMR, POLE, or POLD1 deficient, there is no difference in survival compared to patients with low (<10 mut/Mb) TMB tumors also submitted to ICIs therapy (36). Although such an analysis did not specifically evaluate HRD, it emphasizes that TMB alone should not be considered a biomarker of sensibility to ICIs. Furthermore, the accumulation of genetic errors at the DNA strands’ breaks level leads to a different set of a mutational landscape than DNA sequence alterations that characterize dMMR tumors (37), thus supporting that a high TMB in the presence of HRD may not correlate with the same efficacy seen in MMR or *POLE/POLD1* deficiencies.

Pan-tumor studies have shown that patients with genetic alterations classified as HR deficient frequently present with a high number of small deletions (indels) with flanking microhomology at the breakpoint, in addition to copy number variations (CNVs) (38). Notably, a pan-cancer TCGA analysis demonstrated that the levels of CNVs inversely correlated with a cytotoxic immune signature and clinical benefit from ICIs therapy (39). Moreover, when comparing tumors having a similar oncogenetic driver background but differing with respect to a *BRCA1* or *BRCA2* mutation, there is a significant difference in the levels of CNVs between each of these different HR deficient subtypes, in addition to a distinct set of immunoregulatory genes and ICIs efficacy (40). Conversely to *BRCA2* tumors, those with *BRCA1* deficiency presented with an immunoregulatory infiltrate and a limited response to ICIs. Moreover, another in-depth TCGA analysis also pointed to the coexistence of anti-tumoral immune transcripts downregulation, such as IFN-γ related genes, with the upregulation of immunosuppressive markers related to myeloid tolerogenic cells activity in *BRCA1* mutated breast cancers (41). Altogether, these data suggest that the tumoral HR-related genetic modifications could differentially regulate immune responses.

The molecular mechanisms supporting why CNVs or other specific genetic features associated with HRD mitigate immune responses remain unclear. Speculative hypothesis resides on large-scale mutational alterations leading to protein imbalance that impair tumor signals needed for cytotoxic immune cell infiltration or to deregulation of tumor signaling pathways that ultimately regulate immune cell recruitment (39). For a proper tumor antigen presentation, extensive integrity within the large HLA complex and the whole antigen processing machinery should be met (42). That complexity highlights the various vulnerable points that might lead to a dysfunctional tumor antigen presentation. The presence of CNVs can be associated with impaired antigen presentation owing to proteotoxic stress. Accordingly, the increased flux of unstable wild-type proteins may saturate critical chaperones and the proteasome complex while generating more self-peptides that ultimately place neoantigens at a further competitive disadvantage for loading onto limiting MHC protein (43).

Somatic copy number variation may also hinder tumor antigenic recognition through the downregulation of MHC I molecules. Extensive TCGA analyses demonstrate that loss of heterozygosis (LOH) in any MHC I genetic complex loci frequently accompanies tumors harboring chromosomal instability owing to alterations in cell cycle checkpoint genes such as *TP53*, in addition to HR deficient genes. Furthermore, tumor models with genomic instability frequently evolve with DNA hypermethylation silencing of genes belonging to the antigen presentation through MHC class I pathways (44). It is also noteworthy that a non-linear correlation between HLA-I LOH, TMB, and neoantigen burden has been suggested, such that HLA-I LOH is a frequent immune evasion mechanism in tumors overall, except for those with an either low or high (>30 mut/Mb) TMB, the latter of which are commonly represented by MMRd tumors (45).

In order to leverage neoantigen load and, thus, tumor recognition by immune cells, ongoing prospective studies are now evaluating PARPi added to ICIs in various HR deficient scenarios. Although it is attempting to speculate that further inducing inflammation in a somewhat immune-excluded tumor might restore anti-tumoral immune responses, some concerns may still be set. As aforementioned, neoantigen presentation’s multifaceted and complex processes may hamper tumor recognition despite efforts to enhance immunogenicity by fostering tumor mutations, particularly in settings where at least a non-low tumor mutation burden and neoantigen load predominates. In such conditions, immune-tolerance likely occurs due to multiple coexisting mechanisms such as dysfunctional neoantigen presentation and CD8+ cells exhaustion mediated by cell-cell interactions and other non-ligand-receptor interactions that lead to immune resistance. As such, none of these mechanisms would be reversed by the primary intention of using iPARP to enhance tumor neoantigen load. Furthermore, PARP inhibitors in the presence of HR defects could foster the emergence of subclonal mutations that contribute to establishing intratumor heterogeneity under the pressure of the immunoediting process (46, 47). Indeed, intratumor heterogeneity has also been associated with ICIs resistance (48).

### DDR and PD-L1 Expression

Cancer cells with dysfunction at the DNA strand break repairing apparatus increase the rate of DNA repair basal activity to establish genome stability, particularly in the presence of constant cell proliferation. When molecular cascades featuring the homologous repair system are operating, checkpoint kinase 1 (Chk1) activation can also trigger the STAT1 – STAT3 – IRF1 signaling pathway, inducing PD-L1 expression in tumor cells (49). This model of tumor intrinsic PD-L1 expression, which is dependent on oncogenetic tumor features, has been defined as constitutive to distinguish the so-called acquired expression, in which tumor cells express PD-L1 in response to IFN-γ expression mediated by antitumor lymphocyte activity (50). The DNA repair signaling pathway ATR/Chk1/STAT3 can also upregulate CD47 and, through the engagement of SIRPα, suppress the capacity of antigen-presenting cells (APCs) to phagocytose and cross-present (51).

Concerning the constitutive tumor PD-L1 expression, ICIs may poorly correlate with response and survival, paradoxically predicting less benefit with the anti-PD-1/PD-L1 blockade in some tumors. Although PD-L1 expression is strongly correlated with clinical benefit in non-small cell lung cancer, tumors with EGFR activated-mutations, which can upregulate PD-L1 expression (52), do not derive benefit from ICIs’ treatment (53). Likewise, in the context of PD-L1 expression mediated by HRD, *BRCA1* mutated breast cancer has been demonstrated to have a higher PD-L1 tumor score than *BRCA2* mutated, even though clinical efficacy is inferior (41, 54). Not only do these data support that the PD-L1 expression does not represent a perfect biomarker for ICIs response across all tumor settings, but also suggest that a non-canonical tumor PD-L1 expression (i.e., constitutive) might even associate with mechanisms of immune resistance.

**Figure 1:**
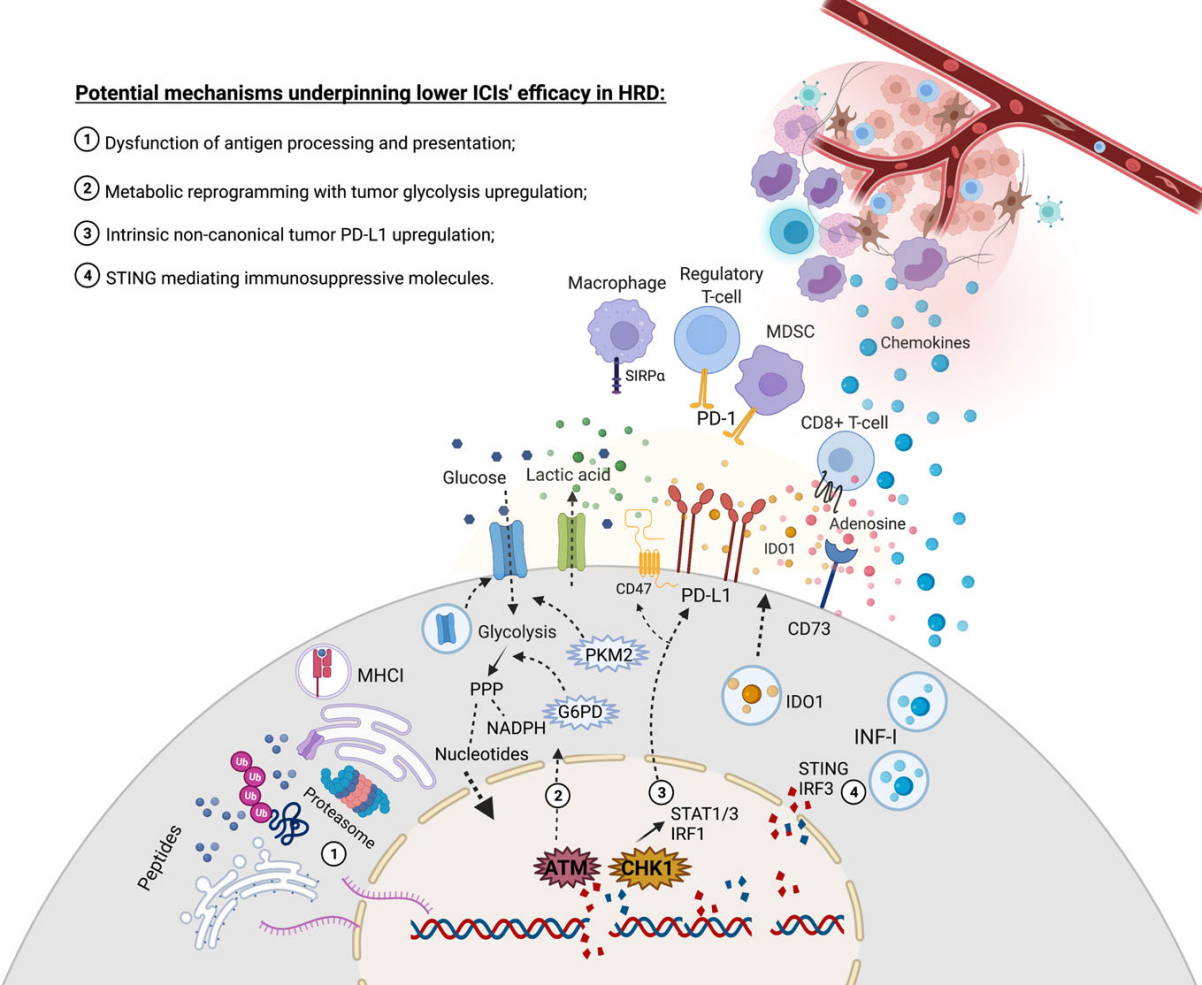
Increased rate of DNA double strands breaks due to deficiency in HR might evolve with molecular events that lead to challenges in restoring immune responses through immune checkpoint inhibitors. (1) DNA double-strand breaks association with CNV and large structural genetic alterations contribute to an increased flux of unstable mRNA and, ultimately, proteins that may saturate critical chaperones and the proteasome complex, thus leading to a dysfunctional tumor antigen presentation. (2) ATM plays a central role in recognizing DNA strand breaks but can also upregulate glycolysis and PPP to replenish nucleotides and NADPH supply for the upcoming anabolic reactions to restore DNA damages. This metabolic regulation might deprive glucose in the tumor microenvironment and export acid lactic, impacting immune responses. (3) *CHK1* is crucial to repair strands breaks but may also activate the STAT1-STAT3-IRF1 signaling pathway that contributes to upregulating PD-L1 expression. (4) Cytosolic DNA censoring can lead to STING-IRF3 production of IFN-I, which might recruit monocytes that will be further exposed to a range of tolerogenic stimuli in the tumor microenvironment. Moreover, the STING signaling pathway might induce IDO1, and the expression of IFN-I might upregulate CD73, thus contributing to producing inhibitory molecules in the tumor microenvironment. Created with BioRender.com.

The HR-driven constitutive PD-L1 expression, which occurs in a non-canonical fashion, irrespective of effector T cells activity, might mitigate ICIs efficacy by hypothetical mechanisms. Firstly, and simplistically, a sufficient lymphocyte infiltration to be restored by ICIs is essential for an effective immune response to take place. Indeed, tumor immune infiltrates (TIL) are a known biomarker predicting clinical efficacy to ICIs in various tumors (55, 56). In this regard, the simple fact of witnessing PD-L1 expression does not guarantee that this results from the positive pressure (i.e., INF-γ driven) of the presence of an immune infiltrate. Secondly, even in the presence of an adequately primed effector immune infiltrate, the constitutive tumor PD-L1 expression fomented by HRD could provide an overwhelming pool of ligands to the PD-1-expressing immune cells that might occupy the tumor microenvironment. Therefore, this could help to polarize immune responses towards a suppressive spectrum, as exemplified by the PD-L1 persistent inducement of FOXP3 expression (FOXP3 ^high^) in PD-1+ T-cells (57), which are characteristically associated with a decreased capacity to reinvigorate into anti-tumoral responses despite ICIs’ activity (58, 59). Lastly, the constitutive expression of PD-L1 may also provide evolutionary metabolic advantages to cancer cells by fostering tumor glycolysis and, in turn, impacting immune cells’ metabolic fate (60). As such, the PD-L1 expression in cancer cells can directly regulate tumor metabolism through Akt/mTOR signaling, independently of the PD-1 engagement, therefore upregulating tumor glycolysis that leads to microenvironment glucose deprivation and lactic acid concentration (**Figure 1**).

### DDR and Metabolic Reprogramming

Tumor cells with DDR defects have a high requirement to restore DNA damage through compensatory pathways. Ataxia-telangiectasia mutated (*ATM*) and DNA-dependent kinases (*DNA-PK*) are crucial proteins to recognize DNA damage and initiate repair signaling cascades. Besides their function in DNA strand-break repair, these proteins can remodel cancer metabolism through upregulation of glucose transporter (*GLUT*) channels and pyruvate kinase M2 (*PKM2*) enzyme, thus fostering tumor glycolysis (61). Hyperactivation of glycolysis is one of the hallmarks of cancers and has been implicated in immune evasion owing to nutrient competition and toxic metabolites accumulation, such as lactic acid (60). Furthermore, ATM activity can also induce glucose-6- phosphate dehydrogenase (*G6PD*) expression, which is fundamental to enable the pentose phosphate pathway (*PPP*) (62). The oxidative PPP generates ribose-5-phosphate, a precursor for nucleotide synthesis, and reduces the potential in the form of NADPH, which is needed for nucleotide biosynthesis and lipogenesis (**Figure 1**). Previous studies also demonstrated that *BRCA1* mutation and PARP1 activity also influence tumor metabolism. The *BRCA1* lack in breast cancer was associated with increased glycolytic metabolism than *BRCA1*-WT (63, 64). Furthermore, it was demonstrated that PARP1 works as a transcriptional coactivator for *PKM2* driving the expression of glycolytic genes (GLUT and LDH) in tumor cells (65). However, the role of metabolic changes induced by *BRCA1* and PARP1 on primary resistance to ICIs remains unknown.

### DDR and STING

A dysfunctional HR status predisposes cancer cells to DNA strands fragmentation in the presence of additional DNA damaging factors, such as radiotherapy, chemotherapy, or PARP inhibitors. Furthermore, DNA instability can occur spontaneously owing to the high tumor cell turn-over coupled with cell cycle checkpoints suppression and enhanced metabolic stress due to tumor metabolism deregulation and microenvironmental hypoxia. This background predisposes to frequent cytosolic DNA exposure in cancer cells. The cytosolic DNA activates the stimulator of IFN genes pathway (*STING* pathway) and *IRF3* activity, thus inducing the transcription of IFN type I and chemoattractive cytokines (CXCL10 and CCL5), which mediates monocytes and neutrophil recruitment in an antigen-independent manner (66). Although type I IFN is a known contributor to T cell priming by inducing MHC I antigen cross-presentation in APCs, there have been growing insights linking STING-IFN molecular pathways to mechanisms mitigating effective immune responses (67, 68). Accordingly, an enhanced baseline STING-IRF3 activity can promote the sustained recruitment of monocytes in response to CXCL10 and CCL5 chemokines, inducing a chronic myelocytic inflammatory infiltrate that could further contribute to establishing an immune tolerogenic state (69). Those constant levels of DNA damage featuring HR deficient tumors can also activate an alternative STING pathway through ATM-TRAF6 driven transcription of TGF-β that promotes protumor M2-like macrophage and Treg cell differentiation, respectively (70). Lastly, the *STING* signaling pathway can also contribute to establishing a tolerogenic tumor microenvironment by inducing immune-suppressive soluble factors. An increase in IDO expression was shown to occur in STING mediated fashion when in the presence of mild tumor antigenicity (71). Moreover, the augmented IFN-α expression has been shown to upregulate the ectonucleotidase CD73 and leverage adenosine production in a tumor microenvironment wherein DDR might be fostering ATP production (72) (**Figure 1**).

## Summary and Future Perspectives

The data summarized in this review suggest that HRD tumors have a differential profile of efficacy and resistance to ICIs’ treatment compared to other dMMR. Each DDR deficient pathway could lead to the emergence of a singular tumor mutational background, but the correlation between such a range of mutational patterns and the response to ICIs remains unclear. Furthermore, various mechanisms potentially impacting immune responses could emerge from the increased DDR pathways activity, which leads to tumor metabolic rearrangements and microenvironmental recruitment of immune-suppressive factors. The TMB status may not be a pan-cancer predictive biomarker for immunotherapy response, and the incorporation of tumor DDR pathways might be necessary for future genomic biomarker refinements. As such, it would be interesting to carry out studies on tumors harboring different defects in DNA repair pathways.

## Author Contributions

SBS and LMC conceived the work. SBS, CW, SW and LMC wrote the manuscript. All authors contributed to the article and approved the submitted version.

## Funding

This study was supported by grant 2020/10960-5, São Paulo Research Foundation (FAPESP).

## Conflict of Interest

LMC reports grants from Novartis and BMS.

The remaining authors declare that the research was conducted without any commercial or financial relationships that could be construed as a potential conflict of interest.

## Publisher’s Note

All claims expressed in this article are solely those of the authors and do not necessarily represent those of their affiliated organizations, or those of the publisher, the editors and the reviewers. Any product that may be evaluated in this article, or claim that may be made by its manufacturer, is not guaranteed or endorsed by the publisher.
